# Vitamin D Deficiency in Primary Sjögren’s Syndrome: Association with Clinical Manifestations and Immune Activation Markers

**DOI:** 10.31138/mjr.33.1.106

**Published:** 2022-03-31

**Authors:** Panagiotis Athanassiou, Clio Mavragani, Lambros Athanassiou, Ifigenia Kostoglou-Athanassiou, Michael Koutsilieris

**Affiliations:** 1 Department of Rheumatology, St. Paul’s Hospital, Thessaloniki, Greece,; 2 Molecular Physiology-Clinical Application Unit, Department of Physiology, Medical School, National and Kapodistrian University of Athens, Athens, Greece,; 3 Department of Rheumatology, Asclepeion Hospital, Voula, Athens, Greece,; 4 Department of Endocrinology, Asclepeion Hospital, Voula, Athens, Greece,; 5 Department of Physiology, Medical School, National and Kapodistrian University of Athens, Athens, Greece

**Keywords:** vitamin D, Sjögren’s syndrome, immunologic markers, histopathological characteristics

## Abstract

Vitamin D is an agent involved in bone and mineral homeostasis. It has been recognized as a potent immunomodulator. It has immune-enhancing properties, and it induces immune tolerance. Vitamin D deficiency has been shown to be related to the development of autoimmune disorders. Vitamin D deficiency has been observed in patients with rheumatoid arthritis (RA) and it has been shown to be related with disease activity. Vitamin D deficiency has also been found in patients with systemic lupus erythematosus (SLE) and it was shown to be related to disease activity and renal involvement. Vitamin D deficiency has also been observed in multiple sclerosis. Vitamin D has been found to act as a supplemental therapeutic agent in multiple sclerosis. Sjögren’s syndrome is a systemic autoimmune disease affecting the exocrine glands, known as an autoimmune epithelitis. The disease has a complex pathogenesis, requiring a genetic background, immune cell activation, and autoantibody production. The disease affects the exocrine glands, lacrimal, and salivary glands leading to ocular and oral dryness. Vitamin D levels have been measured in patients with Sjögren’s syndrome and an association was observed between low vitamin D levels, peripheral neuropathy and the presence of lymphoma. In other cohorts, such as a Turkish cohort, vitamin D deficiency was observed in patients with Sjögren’s syndrome. The aim is to measure serum vitamin D levels in consecutive patients with primary Sjögren’s syndrome and investigate the relationship between vitamin D levels and the presence of immunologic markers, clinical, serological, and histopathological characteristics.

## INTRODUCTION

Sjögren’s syndrome is a systemic auto-immune disease affecting the exocrine glands,^1,2^ known as autoimmune epithelitis,^3,4^ given the presence of lymphocytic infiltrates around affected epithelia. The disease is characterized by a complex pathogenesis as a genetic background is required along with immune cell activation and autoantibody production. The disease affects the exocrine glands mainly lacrimal and salivary glands leading to generation of oral and ocular dryness known as “sicca syndrome”^5,6^, while systemic manifestations are not uncommon. Hashimoto’s thyroiditis is commonly observed in patients with Sjögren’s syndrome.^5^ Loviselli et al. studied thyroid function parameters and thyroid antibodies in a cohort of patients with primary and secondary Sjögren’s syndrome. They found higher prevalence of thyroid antibodies in patients with Sjögren’s syndrome, which was more pronounced in primary Sjögren syndrome.^7^ It has been suggested that Sjögren’s syndrome and autoimmune thyroid disease may be the two sides of the same coin.^8^

Vitamin D is an agent involved in the regulation of bone/mineral metabolism^9^ as well as in immune pathways.^10–12^ It has immune-enhancing properties,^13,14^ and is also known to induce immune tolerance.^15–17^ Vitamin D deficiency has been related to the development and disease activity of autoimmune disorders,^18,19^ including rheumatoid arthritis,^20,21^ systemic lupus erythematosus,^22^ and multiple sclerosis.^23^ In the latter group, vitamin D supplementation may act as a supplemental therapeutic agent.^24^ Agmon-Levin et al.^25^ measured vitamin D levels in a cohort of patients with Sjögren’s syndrome, and they found an association between low vitamin D levels, peripheral neuropathy, and the presence of lymphoma, while in a cohort of Turkish patients with Sjögren’s, lower vitamin D levels were observed^26^ and in a cohort of Indian patients low vitamin D levels were related to a high risk for high lip grading and rheumatoid factor positivity.^27^

## AIM

The aim was to measure serum vitamin D levels in consecutive patients with Sjögren’s syndrome and investigate the relationship between vitamin D levels and the presence of immunologic markers, clinical, serological, and histopathological characteristics.

## PATIENTS AND METHODS

For our study, 25(OH)D_3_ levels will be retrospectively measured in stored sera from consecutive patients with primary Sjögren’s syndrome (SS) in the “Molecular Physiology-Clinical Application Unit”, Department of Physiology, National and Kapodistrian University of Athens as well as in prospectively collected sera from the Department of Rheumatology St. Paul’s Hospital, Thessaloniki, Greece and Department of Rheumatology, Asclepeion Hospital, Voula, Athens, Greece following written consent to participate in the study. All primary SS subjects participating in the study fulfil the revised international criteria for the classification of primary SS.^28^ Healthy individuals of similar age and sex distribution to the patients with primary SS will be also included. Exclusion criteria for all participants include pregnancy, age <18 years, and renal dysfunction (serum creatinine levels >3 mg/dl, creatinine clearance <30 ml/minute).

Demographic data, clinical features, and therapeutic regimens will be recorded in all participating patients and controls. Demographic data including age, sex, and BMI will be recorded. Clinical manifestations including the presence of subjective and objective oral and ocular dryness (documented by unstimulated salivary flow rates and Schirmer’s test/Rose Bengal staining, respectively); dry cough; dyspareunia; fever; arthralgias; arthritis; carpal tunnel syndrome (documented by physical examination and nerve conduction studies); Raynaud’s phenomenon; lymphadenopathy; splenomegaly; purpura; pulmonary involvement (small airway disease or interstitial lung disease documented by pulmonary function tests and high resolution computed tomography scans); pleuritis; pericarditis; renal involvement including interstitial nephritis (documented by urine-specific gravity <1.010 or pH >5.5 on at least two consecutive measurements after fluid restriction) and glomerulonephritis documented by renal biopsy; liver involvement (documented by liver biopsy showing changes compatible with primary biliary cirrhosis in the setting of increased liver enzymes or anti-mitochondrial antibodies); peri-epithelial disease (defined as peribronchial, interstitial nephritis, autoimmune cholangitis); myositis (documented by muscle biopsy in the setting of increased aldolase or creatinophosphokinase); peripheral neuropathy (documented by nerve conduction studies in patients with clinical symptoms or signs suggestive of neuropathy); central nervous involvement; lymphoma (documented by biopsy) will be recorded. Sjögren’s syndrome disease activity index (ESSDAI) will be determined.^29^

25(OH)D_3_ will be measured by an electrochemiluminescence binding assay (Elecsys Vitamin D total, for cobas e 411 analyser, Roche Diagnostics GmbH, Mannheim, Germany). The assay is based on a competition principle. In summary, after a first incubation, so that the bound 25(OH)D could be released from the vitamin D binding protein, a second incubation is performed. During the second incubation, the pre-treated sample is incubated with a ruthenium labelled vitamin D binding protein. Thus, a complex is formed between 25(OH)D and the ruthenium labelled vitamin D binding protein, the ruthenylated vitamin D binding protein. Thereafter, a third incubation is performed. During the third incubation, streptavidin microparticles and vitamin D labelled with biotin are added, and unbound ruthenium labelled vitamin D binding proteins become occupied. A complex consisting of the ruthenylated vitamin D binding protein and the biotinylated vitamin D is formed and it is bound to the solid phase of the assay via interaction of biotin and streptavidin. The reaction mixture is then aspirated into the cell where the microparticles are magnetically captured onto the surface of an electrode. Unbound substances are then removed. Application of a voltage to the electrode then induces a chemiluminescent emission, which is detected by a photomultiplier. The sensitivity of the assay is 10.03 nmol/L. The within run CV of the assay ranges from 3.1% at 70.0 nmol/L to 7.8% at 16.9 ng/ml.

### Statistical evaluation

Statistical analysis will be performed by SPSS v.21 package. Two-group comparisons of continuous data will be assessed using t-tests, or the Mann-Whitney test, when the data do not have a normal distribution. Comparisons between groups will be performed by Fisher’s exact two tailed test and Mann Whitney test. Difference is considered statistically significant if p<0.05.

## SIGNIFICANCE

Should vitamin D levels found to be associated with the presence of specific immunologic markers in patients with Sjögren’s syndrome, vitamin D substitution may be considered, and underlying mechanisms will be further explored.

## References

[1] Mavragani CP Nezos A Moutsopoulos HM. New advances in the classification, pathogenesis and treatment of Sjögren’s syndrome. Curr Opin Rheumatol Sep 2013;25(5):623–9. 23846338 10.1097/BOR.0b013e328363eaa5

[2] Mavragani CP Moutsopoulos HM. Sjögren’s syndrome: Old and new therapeutic targets. J Autoimmun 06 2020;110:102364. 31831255 10.1016/j.jaut.2019.102364

[3] Ogawa Y Takeuchi T Tsubota K. Autoimmune Epithelitis and Chronic Inflammation in Sjögren’s Syndrome-Related Dry Eye Disease. Int J Mol Sci Oct 30 2021;22(21). 10.3390/ijms222111820 PMC8584177 34769250

[4] Colafrancesco S Barbati C Priori R Putro E Giardina F Gattamelata A Maladaptive autophagy in the pathogenesis of autoimmune epithelitis in Sjögren’s Syndrome. Arthritis Rheumatol Nov 08 2021;doi:10.1002/art.42018. 34748286

[5] Mavragani CP Skopouli FN Moutsopoulos HM. Increased prevalence of antibodies to thyroid peroxidase in dry eyes and mouth syndrome or sicca asthenia polyalgia syndrome. J Rheumatol Aug 2009;36(8):1626–30. 19605678 10.3899/jrheum.081326

[6] Mavragani CP Moutsopoulos HM. Sicca syndrome following immune checkpoint inhibition. Clin Immunol 08 2020;217:108497. 32531346 10.1016/j.clim.2020.108497

[7] Loviselli A Mathieu A Pala R Mariotti S Cau S Marongiu C Development of thyroid disease in patients with primary and secondary Sjögren’s syndrome. J Endocrinol Invest Oct 1988;11(9):653–6. 3221041 10.1007/BF03350206

[8] Anaya JM Restrepo-Jiménez P Rodríguez Y Rodríguez-Jiménez M Acosta-Ampudia Y Sjögren’s Syndrome and Autoimmune Thyroid Disease: Two Sides of the Same Coin. Clin Rev Allergy Immunol Jun 2019;56(3):362–74. 30187363 10.1007/s12016-018-8709-9

[9] Holick MF. Sunlight and vitamin D for bone health and prevention of autoimmune diseases, cancers, and cardiovascular disease. Am J Clin Nutr Dec 2004;80(6 Suppl):1678s–88s. 15585788 10.1093/ajcn/80.6.1678S

[10] Prietl B Treiber G Pieber TR Amrein K. Vitamin D and immune function. Nutrients Jul 5 2013;5(7):2502–21. 23857223 10.3390/nu5072502 PMC3738984

[11] Aranow C. Vitamin D and the immune system. J Investig Med. Aug 2011;59(6):881–6. 10.231/JIM.0b013e31821b8755 PMC3166406 21527855

[12] Sassi F Tamone C D’Amelio P. Vitamin D: Nutrient, Hormone, and Immunomodulator. Nutrients Nov 2018;10(11):1656. 10.3390/nu10111656 PMC6266123 30400332

[13] Bae M Kim H. Mini-Review on the Roles of Vitamin C, Vitamin D, and Selenium in the Immune System against COVID-19. Molecules Nov 16 2020;25(22):5346. 10.3390/molecules25225346 PMC7696052 33207753

[14] Kumar R Rathi H Haq A Wimalawansa SJ Sharma A. Putative roles of vitamin D in modulating immune response and immunopathology associated with COVID-19. Virus Res Jan 15 2021;292:198235. 33232783 10.1016/j.virusres.2020.198235 PMC7680047

[15] Badenhoop K Kahles H Penna-Martinez M. Vitamin D, immune tolerance, and prevention of type 1 diabetes. Curr Diab Rep Dec 2012;12(6):635–42. 22976537 10.1007/s11892-012-0322-3

[16] Chirumbolo S. The role of vitamin D towards immune tolerance in white adipose tissue (WAT). Endocr Metab Immune Disord Drug Targets 2015;15(4):277–87. 26127005 10.2174/1871530315666150701113811

[17] Wei R Christakos S. Mechanisms Underlying the Regulation of Innate and Adaptive Immunity by Vitamin D. Nutrients Sep 24 2015;7(10):8251–60. 26404359 10.3390/nu7105392 PMC4632412

[18] Harrison SR Li D Jeffery LE Raza K Hewison M. Vitamin D, Autoimmune Disease and Rheumatoid Arthritis. Calcif Tissue Int Jan 2020;106(1):58–75. 31286174 10.1007/s00223-019-00577-2 PMC6960236

[19] Illescas-Montes R Melguizo-Rodríguez L Ruiz C Costela-Ruiz VJ. Vitamin D and autoimmune diseases. Life Sci Aug 2019;233:116744. 31401314 10.1016/j.lfs.2019.116744

[20] Ishikawa LLW Colavite PM Fraga-Silva TFC Mimura LAN França TGD Zorzella-Pezavento SFG Vitamin D Deficiency and Rheumatoid Arthritis. Clin Rev Allergy Immunol Jun 2017;52(3):373–88. 27484684 10.1007/s12016-016-8577-0

[21] Kostoglou-Athanassiou I Athanassiou P Lyraki A Raftakis I Antoniadis C. Vitamin D and rheumatoid arthritis. Ther Adv Endocrinol Metab Dec 2012;3(6):181–7. 23323190 10.1177/2042018812471070 PMC3539179

[22] Shoenfeld Y Giacomelli R Azrielant S Berardicurti O Reynolds JA Bruce IN. Vitamin D and systemic lupus erythematosus - The hype and the hope. Autoimmun Rev Jan 2018;17(1):19–23. 29108830 10.1016/j.autrev.2017.11.004

[23] Van Amerongen BM Dijkstra CD Lips P Polman CH. Multiple sclerosis and vitamin D: an update. Eur J Clin Nutr Aug 2004;58(8):1095–109. 10.1038/sj.ejcn.1601952 15054436

[24] van Amerongen BM Feron F. Effect of high-dose vitamin D3 intake on ambulation, muscular pain and bone mineral density in a woman with multiple sclerosis: a 10-year longitudinal case report. Int J Mol Sci Oct 19 2012;13(10):13461–83. 23202962 10.3390/ijms131013461 PMC3497336

[25] Agmon-Levin N Kivity S Tzioufas AG López Hoyos M Rozman B Efes I Low levels of vitamin-D are associated with neuropathy and lymphoma among patients with Sjögren’s syndrome. J Autoimmun Sep 2012;39(3):234–9. 22835660 10.1016/j.jaut.2012.05.018

[26] Erten Ş Şahin A Altunoğlu A Gemcioğlu E Koca C. Comparison of plasma vitamin D levels in patients with Sjögren’s syndrome and healthy subjects. Int J Rheum Dis Jan 2015;18(1):70–5. 24467766 10.1111/1756-185X.12298

[27] Sandhya P Mahasampath G Mashru P Bondu JD Job V Danda D. Vitamin D Levels and Associations in Indian Patients with Primary Sjögren’s Syndrome. J Clin Diagn Res Sep 2017;11(9):oc33–oc36. 10.7860/JCDR/2017/28493.10697 PMC5713779 29207757

[28] Shiboski CH Shiboski SC Seror R Criswell LA Labetoulle M Lietman TM 2016 American College of Rheumatology/European League Against Rheumatism classification criteria for primary Sjögren’s syndrome: A consensus and data-driven methodology involving three international patient cohorts. Ann Rheum Dis Jan 2017;76(1):9–16. 27789466 10.1136/annrheumdis-2016-210571

[29] Seror R Ravaud P Bowman SJ Baron G Tzioufas A Theander E EULAR Sjögren’s syndrome disease activity index: development of a consensus systemic disease activity index for primary Sjögren’s syndrome. Ann Rheum Dis Jun 2010;69(6):1103–9. 19561361 10.1136/ard.2009.110619 PMC2937022

